# Tuning Barrier Properties of Low-Density Polyethylene:  Impact of Amorphous Region Nanostructure on Gas Transmission Rate

**DOI:** 10.3390/molecules29204950

**Published:** 2024-10-19

**Authors:** Marta Safandowska, Cezary Makarewicz, Artur Rozanski

**Affiliations:** Centre of Molecular and Macromolecular Studies, Polish Academy of Sciences, Sienkiewicza 112, 90-363 Lodz, Poland; cezary.makarewicz@cbmm.lodz.pl

**Keywords:** LDPE blends, polymer structure, oxygen permeation

## Abstract

This work focused on determining the factors that are of key importance in the oxygen barrier properties of low-density polyethylene (LDPE). It has been shown that, depending on the type and amount of the low-molecular-weight compound (tetracosane, paraffin wax, paraffin oil) introduced into the LDPE matrix, it can contribute to the improvement or deterioration of barrier properties. Tetracosane and paraffin wax incorporated into the LDPE matrix caused a reduction in oxygen permeability parameters compared to neat polyethylene. As their content increased, the barrier properties of the samples towards oxygen also increased. A completely opposite effect was achieved with paraffin oil. The results of comprehensive studies provide evidence that in the case of LDPE blends, two mechanisms are responsible for changing/controlling their transport properties. The first mechanism is associated with changes in the molecular packing in the interlamellar amorphous regions, while the second is related to the crystallinity of the samples. In cases where there are no changes in crystallinity, the density of the amorphous phase becomes the decisive factor in barrier properties, as clearly shown by results assessing chain dynamics.

## 1. Introduction

Packaging made from polyethylene (PE) is a substantial component of the worldwide plastic packaging industry. It is estimated that profits from this sector in the US plastic packaging market have currently exceeded USD 130 billion [1,2]. Low-density polyethylene (LDPE) was the first plastic to come into common commercial use in packaging in the late 1940s [3]. Thanks to its high versatility and adaptability to various production processes and applications, LDPE now accounts for approximately 16% (15 million tons) of the annual global plastic production. LDPE has high clarity, is chemically inert, has good impact strength, and has excellent tear and stress crack resistance. It is widely used in packaging, such as foils, trays, and plastic bags for both food and non-food purposes, as well as protective film on paper, textiles, and other plastics [4,5,6,7]. Although other materials are currently being proposed as alternatives to plastics [8], synthetic polymers still remain the preferred choice for packaging materials, mainly due to their low cost, ease of use, and durability [9]. At the same time, these properties have led to the indiscriminate use of plastics, which unfortunately now represent a large portion of the solid waste produced globally. It is therefore worthwhile to design packaging in such a way as to extend the shelf life of perishable goods and to ensure that the packaging is recyclable [10]. The non-polarity of polyethylene hydrocarbon chains confers good moisture barrier properties, reflected in a low water vapor transmission rate (WVTR) [11]. Nonetheless, LDPE displays a high oxygen transmission rate (OTR), which is detrimental for packaging perishable items such as raw food, cosmetics, and medicines [4,7]. The diffusion of gas across a film is dependent on its structure, thickness, surface area, and temperature of measurement. In the absence of cracks or other imperfections, the primary mechanism of gas and water vapor flow through a polymeric film is the solution-diffusion mechanism [12,13].

Low-density polyethylene belongs to the class of semicrystalline polymers, which, during solidification, exhibit the ability to form a specific structure where crystals are separated by disordered, amorphous layers. The lamellar crystals, characterized by a unique arrangement of macromolecules, effectively block the passage of penetrating substances, even those with small particle sizes [14]. As a result, the transport properties primarily depend on the content and microstructure of the crystalline component, as well as on the nano-/microstructure of the amorphous component. The relationship between structure and gas barrier properties in semicrystalline polymers has been discussed for decades [4,11,15,16,17]. To date, some studies have suggested that increasing crystallinity contributes monotonically to extending the tortuous diffusion path and reducing the sorption capacity by decreasing the content of the amorphous phase, while in others, this relationship has not been as straightforward [13,18]. An essential point to underscore is the tendency to disregard the amorphous phase, despite its critical role. This oversight hampers the comprehensive understanding and effective optimization of final properties of polymer materials. At the same time, aiming for an increase in material crystallinity is not always the goal, as it may lead to undesirable changes in thermomechanical properties, for example, a reduction in the elasticity or flexibility of the polymer. In our previous researches, focusing on enhancing oxygen barrier properties of polylactide (PLA) [19] and high-density polyethylene (HDPE) [20], we have underscored the critical role played by the nano-/microstructure of the amorphous phase, particularly the molecular packing efficiency within this region. The papers have demonstrated the usefulness of low-molecular-weight compounds in increasing the packing density of the amorphous phase in both PLA and HDPE, and consequently in improving their oxygen barrier properties.

This work extends our strategy of modifying the non-crystalline regions of semicrystalline polymers. Regarding LDPE, the incorporation of low-molecular-weight compounds into the polymer matrix may serve as a key factor in modifying the packing density of the amorphous phase. Given LDPE’s typically higher proportion of amorphous regions compared to HDPE, this could potentially bring about favorable alterations in transport properties. However, the decisive factor in improving barrier properties while maintaining other properties of LDPE would likely depend on various factors such as the compatibility of the added modifier molecules with the LDPE matrix, the extent of interaction between the additive and LDPE, and the resulting changes in the microstructure of the polymer. Herein, we report a series of studies focused on evaluating the impact of three modifiers (tetracosane, paraffin wax, and paraffin oil), each with subtly different molar masses and solubility parameters, on the microstructure, chain mobility, and gas transport characteristics of LDPE films. The inclusion of low-molecular-weight additives enables the alteration of the structure of semicrystalline polymers like polylactide (PLA), high-density polyethylene (HDPE) [21], or polypropylene (PP) [22], resulting in property adjustments. In our recent papers [19,20], we showed that by selectively introducing low-molecular-weight modifiers into the amorphous regions of PLA or HDPE, the efficiency of packing macromolecules in these regions and the free volume were changed, thereby determining the transport properties of the polymers.

Considering a novel strategy in designing polymer systems for packaging purposes is crucial to produce films with good oxygen barrier properties under broad usage conditions that are engineered for sustainability. The proposed approach to enhance the transport properties of low-density polyethylene is primarily easy to implement in processing. Furthermore, employing modifiers with similar chemical structures to polyethylene could play a pivotal role in the recycling of such materials.

## 2. Results and Discussion

In an attempt to change the microstructure of low-density polyethylene (LDPE) and thus improve the oxygen barrier properties, in this work, LDPE blends with three different additives (tetracosane, paraffin wax, paraffin oil) were prepared.

Firstly, by tracking the weight loss observed in (thermogravimetric analysis) TGA curves (Figure S1, Supplementary Materials), which corresponds to the volatilization of low-molecular-weight additives, their concentration in LDPE blends was determined (Table 1). Considering the expected loss of additives during melt blending process, the initial concentration of additives was intentionally set to be at least 5% higher than the resulting concentration.

**Figure 1:**
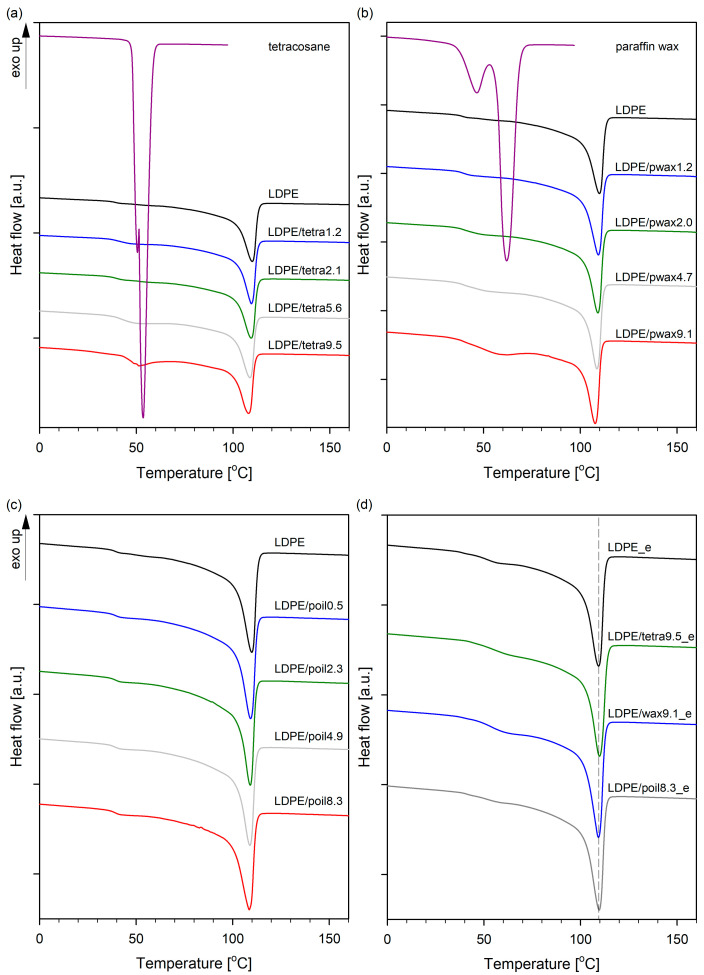
DSC curves of LDPE systems with tetracosane (**a**), paraffin wax (**b**), paraffin oil (**c**), and after modifier removal (**d**).

A series of differential scanning calorimetry (DSC) tests with a constant heating rate were performed to assess the influence of type and concentration of additive on the thermal transitions of LDPE. Figure 1 depicts the DSC melting curves of pure components and the blends. The melting temperatures (*T*_m_) and degrees of crystallinity (*X*_C_) are summarized in Table 1.

In the case of pure LDPE, only one endothermic peak, related with the melting transition, was observed at 109.4 °C. Tetracosane exhibited two endothermic peaks at 50.5 and 53.5 °C, while paraffin wax displayed peaks at 42.9 and 59.7 °C. The first peak was associated with a solid–solid transition, while the second peak denoted the solid–liquid transition [23,24]. The enthalpies during the melting of tetracosane, paraffin wax, and LDPE was calculated as 255.8, 190.7, and 116.6 J/g, respectively. The melt transition temperatures of the series of LDPE/modifier systems decreased proportionally from 0% (109.4 °C) to ~9.5% modifier (~107.8 °C). This indicates that the used low-molecular-weight additives acted as plasticizing agents for low-density polyethylene. Since the additives melted at lower temperatures than LDPE or were in a molten state at room temperature (paraffin oil), the unmolten LDPE crystals were surrounded by molten molecules of tetracosane, paraffin wax, or paraffin oil. As a result, melting point depression was observed, which is in accordance with the Flory–Huggins theory, which that the additives act like solvents [25]. It is also worth noting that in all blends of low-density polyethylene with paraffin oil, only one endothermic peak was observed, similar to pure LDPE. Conversely, for systems containing tetracosane or paraffin wax as additives, higher concentrations of these additives (from 4.7%) resulted in an additional peak on the DSC curves, the intensity of which increased with the modifier content in the LDPE blend. The appearance of an extra peak in the DSC curves was associated with a solid–liquid transition of additives molecules and, thus, indicates the phase separation of the modifier in these systems. The distribution of additive in the polymer matrix of LDPE/tetra5.6, LDPE/tetra9.5, LDPE/pwax4.7, and LDPE/pwax9.1 was therefore heterogeneous; the modifier was partly dispersed at a molecular level in the amorphous regions and partly formed modifier-rich domains. For polyethylene and paraffin oil, completely miscible systems were obtained in the full range of modifier concentrations. The miscibility of the systems is important from the point of view of their further properties, especially in the context of thermomechanical properties, free volume, and, consequently, gas barrier properties. The degree of crystallinity (*X*_C_) in LDPE/modifier systems was influenced by the combination of additive with LDPE. In blends with tetracosane and paraffin wax containing the modifier in an amount not exceeding 4%, the degree of crystallinity remained practically unchanged relative to pure polymer. For blends with 5.6% tetracosane and 4.7% paraffin wax, or more, the degree of crystallinity of the films displayed an upward trend (by a maximum of 2–4%) when compared to the pure LDPE. When LDPE was combined with paraffin oil, the crystallinity remained consistent across varying concentrations of paraffin oil. It can be seen that the *X*_c_ stayed in LDPE/poil blends at around 39% throughout the entire range of paraffin oil concentrations tested. The rise in crystallinity of LDPE in blends as tetracosane and wax content increased was evidenced by an increase in melting enthalpy. Some authors dealing with the thermal properties of polyethylene blends, especially those incorporating various paraffin waxes, believe that the reason for such behavior of the blends is the inclusion of short and linear wax chains into the crystal lattice during crystallization. This phenomenon is commonly known as the cocrystallization effect. For cocrystallization to occur, appropriate criteria must be met. Arnal et al. [26] pointed out that in the case of blends of linear and branched polyethylene blends, only those chain fractions with linear crystallizable sequences of similar lengths are able to cocrystallize. Gumede et al. [27,28], based on additional, successive self-nucleation and annealing (SSA) measurements, also found that wax chains could cocrystallize solely with a small fraction of lamellae formed by linear segments of LLDPE chains with the highest short-chain branch contents. The phenomenon of cocrystallization between LDPE chains and the used low-molecular-weight (tetracosane, paraffin wax, paraffin oil) additives seems unlikely. Particularly, after the removal of the additives through solvent extraction, the melting temperature peak of LDPE blends shifted towards the value characteristic of low-density polyethylene, and the degree of crystallinity also aligned with that of pure polymer (Figure 1d). Considering the above, it became evident that LDPE was plasticized by the additives with the changes being temporary in nature. Analogous effects were seen by us in the previously examined systems: polylactide/triethyl citrate [19], high-density polyethylene/wax [20,21], polypropylene/wax [29], and others [22]. However, it is worth noting that the interactions are highly dependent on the composition of the blend and the thermodynamic interactions between polymer and additive. Gumede et al. [28], using the Flory–Huggins theory, showed that the interactions of linear low-density polyethylene (LLDPE) with waxes were strongly nonlinear as a function of composition. Wax acts as a solvent for LLDPE, but its impact is complex and greatly varies with composition.

To gain deeper insight into the structure of the prepared LDPE/additive systems and assess the influence of the additive (both type and concentration), X-ray diffraction measurements were performed. The analysis of wide-angle X-ray scattering (WAXS) patterns for LDPE blends revealed that the crystalline structure of LDPE was unaffected by the incorporation of tetracosane, paraffin wax, or paraffin oil molecules.

**Figure 2:**
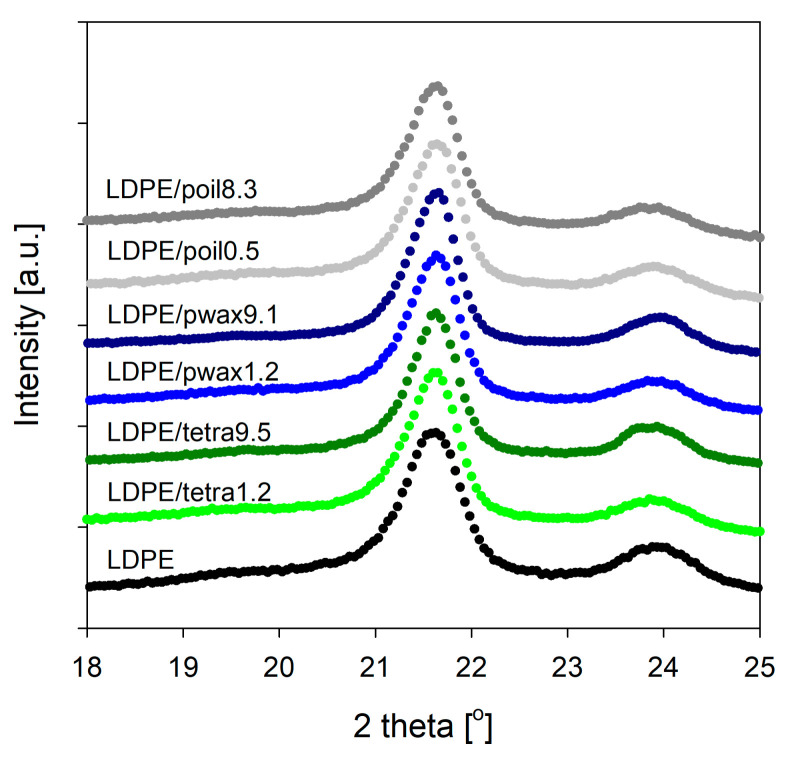
The diffraction patterns of pure LDPE and selected LDPE blends with tetracosane, paraffin wax, and paraffin oil.

Two sharp diffraction peaks appeared in the WAXS pattern of pure LDPE at 21.6° and 23.9°, which were attributed to the (110) and (200) crystallographic planes of the orthorhombic polyethylene unit cell [30] (Figure 2). Analogous diffraction peaks could be distinguished in the WAXS patterns obtained for LDPE/additive blends (Figure 2). This strongly suggests that with the incorporation of low-molecular-weight compounds, the characteristic orthorhombic crystal structure was maintained, signifying that the intrinsic crystal structure of LDPE remained unchanged. On the other hand, compared with pure LDPE, some from the LDPE/modifier systems exhibited slightly larger characteristic intensity, implying that the presence of low-molecular-weight modifiers might enhance LDPE’s crystallinity, consistent with the aforementioned DSC analysis. The greatest increase in intensity was recorded for blends of polyethylene with paraffin wax, particularly for systems containing 4.7 and 9.1% wax. This finding could be connected to the enhanced crystallinity of polyethylene, but it may have also been influenced by the presence of separated additive in the blend. As tetracosane did not display diffraction peaks within the range of 2θ scattering angles 15–25°, and paraffin wax showed the same type of reflections as polyethylene [31,32], the study was extended to include the small-angle X-ray scattering (SAXS) techniques.

**Figure 3:**
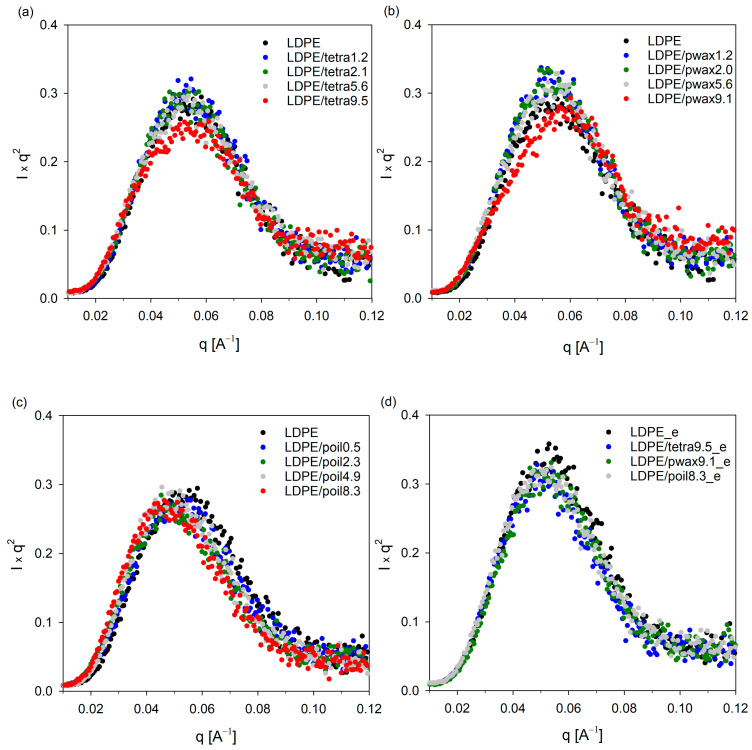
SAXS patterns of LDPE blends with tetracosane (**a**), paraffin wax (**b**), paraffin oil (**c**), and for selected samples after extraction process; samples are marked with _e (**d**).

Figure 3 depicts the Lorentz-corrected SAXS scattering profiles for pure LDPE and the LDPE/additive blends. The one-dimensional integral SAXS curves for all studied systems revealed a single, prominent broad peak that represented the scattering from lamellar stacks. Assuming a two-phase model with an alternately stacked structure of crystalline and amorphous layers, the long periods were estimated from the maximum value of the scattering vector q_max_ using Bragg’s law and are listed in Table 2.

Both from the curves shown in Figure 3a–c and the obtained LP values, it is evident that with the increasing concentration of the modifier in the blend, the LP value increased, as manifested by a shift in the peak position towards lower values. The highest increase in LP was recorded for samples containing paraffin oil; additionally, no changes in crystallinity were noted for these samples. This implies that the introduced paraffin oil molecules, which were preferentially located in the amorphous phase regions, led to its swelling and an increase in the LP value. For the other two additives, tetracosane and paraffin wax, a slight increase in LP was recorded only for samples containing the additives in amounts not exceeding 4%. Further increases in the concentration of these additives in the polyethylene blend had practically no effect on the LP values. This suggests that at higher concentrations, the amorphous phase of LDPE was unable to accommodate more molecules of the additives, tetracosane or paraffin wax, leading to their phase separation, which was confirmed by calorimetric tests. Note that SAXS scattering profiles for the LDPE systems containing 9.5% tetracosane and 9.1% paraffin wax showed broadening. To verify whether the SAXS signal could originate from cocrystals formed between the additive and LDPE, selected blends were evaluated using SAXS technique after modifier extraction. Figure 3d clearly shows that after the extraction process, the scattering pattern of LDPE/tetra9.5_e and LDPE/pwax9.1_e became similar to that of the pure polymer sample, and the long period value also returned to the characteristic value for pure polyethylene. From the DSC studies, presented above, it is clear that these systems exhibited a second peak on the thermograms in addition to the polyethylene melting peak, indicating the melting of the separated modifier fraction. The slight increase in crystallinity in these samples could indicate lamella thickening. However, as observed in the WAXS profiles, the positions and half-widths of individual reflections remained unchanged. Only a slight increase in signal intensity, consistent with the increase in crystallinity, was noted. FTIR spectra can also help determine whether the introduction of the modifier into the low-density polyethylene matrix results in changes only in the amorphous regions or if it also affects the crystalline structure.

**Figure 4:**
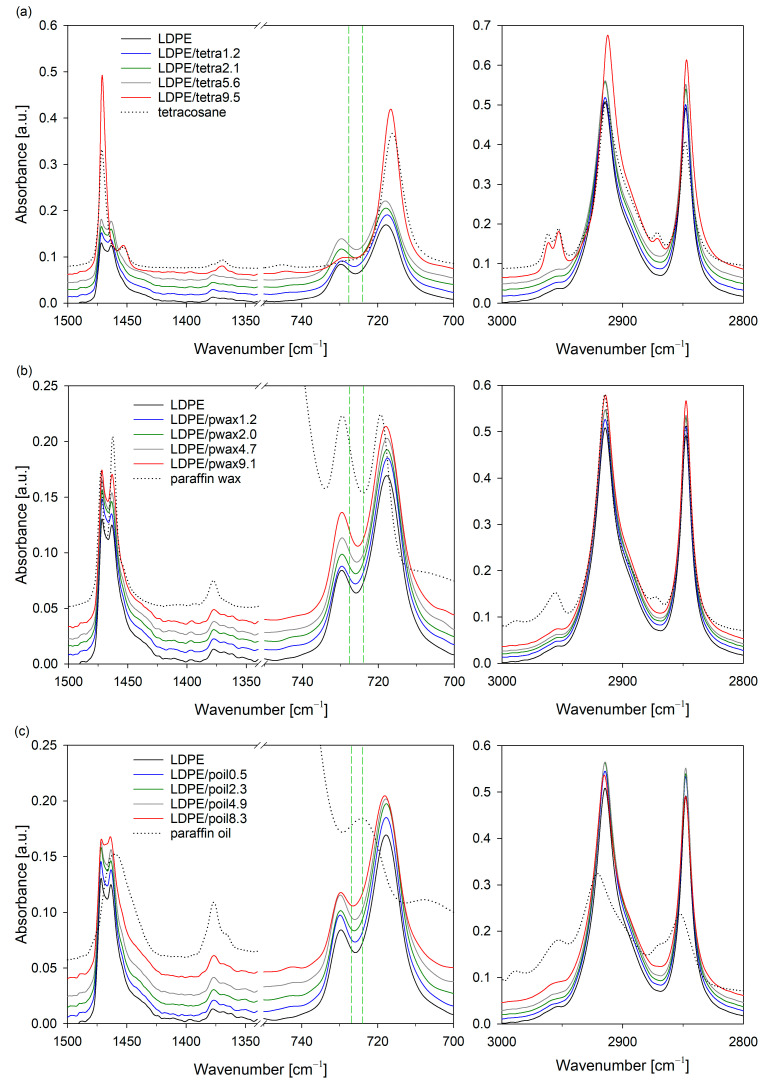
FTIR-ATR spectra of LDPE systems with tetracosane (**a**), paraffin wax (**b**), and paraffin oil (**c**).

Infrared spectroscopy is a non-destructive, micro-analytical technique that is used to check chemical constituents, the configuration of the macromolecules, and also the relationships among the chains (morphology). Figure 4 shows the FTIR spectra of the LDPE/additive blend films and pure components of these systems (LDPE, tetracosane, paraffin wax, paraffin oil). The signals in the ranges of 3000–2800, 1500–1340, and 750–700 cm^−1^ could be clearly assigned to the characteristic peaks of low-density polyethylene [33,34,35]. The vibration band located at 735–705 cm^−1^ corresponded to the CH_2_ rocking deformation in the plane (per rotation) of the connections in the methylene group; 1480–1450 cm^−1^ to bending deformation of CH_3_ and CH_2_; and 1390–1340 cm^−1^ to the balance-type/symmetric deformation of the CH_3_ groups [36]. The signal at 2870–2800 cm^−1^ was related to the symmetrical stretching of CH_2_, while the vibrational band at 2980–2870 cm^−1^ referred to the asymmetric stretching of CH_2_ [37].

The characteristic vibrations of polyethylene could be observed in nearly each prepared LDPE blend, regardless of whether they contained tetracosane, paraffin wax, or paraffin oil. The only exception was the sample containing 9.5% tetracosane. In the spectrum of LDPE/tetra9.5, absorption peaks characteristic of tetracosane (2970–2940, 2875–2865, 1480–1440, and 720–710 cm^−1^) were prominently visible, confirming the phase separation phenomenon in this system.

Extensive research on the molecular structure of polyethylene using infrared spectroscopy has enabled the appropriate assignment of trans-trans and gauche conformations, and as a result, the identification of ordered and less ordered regions [33]. It is well established that the ordered fractions in the orthorhombic modification give a doublet in the rocking (735–705 cm^−1^) and bending regions (1480–1450 cm^−1^) due to the interaction between two chains in the unit cell. It turn, the amorphous fractions of PE can be monitored with the methylene rocking bands near 725 cm^−1^ [33]. Taking the aforementioned points into account and based on a detailed analysis of the IR spectra, it can be concluded that the modifier, irrespective of type and concentration, did not affect the crystal structure of the polyethylene. In the IR spectra corresponding to the crystal structure wavenumbers, peak positions remained largely unchanged. However, for all additives, as their concentration in the LDPE blend increased, a shift of the band near 725 cm^−1^ towards lower values was noticeable. These findings clearly indicate that changes occurred only within the amorphous phase due to the presence of modifier molecules.

**Figure 5:**
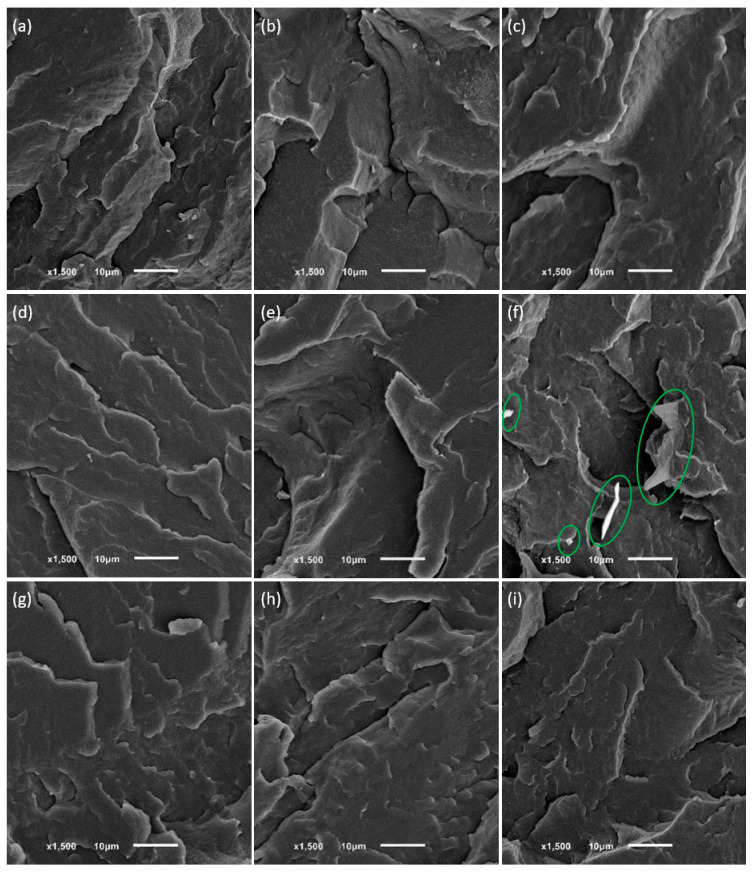
SEM images for the freeze-fractured surfaces of the pure LDPE (**a**) and LDPE blends with paraffin oil: 0.5% (**b**) and 8.3% (**c**); tetracosane: 1.2% (**d**), 5.6% (**e**), and 9.5% (**f**); and paraffin wax: 1.2% (**g**), 4.7% (**h**), and 9.1% (**i**) films. Green circles indicate objects resulting from the phase separation process.

The DSC and FTIR measurement results indicated that phase separation might have occurred in blends of LDPE with either tetracosane or paraffin wax modifiers. Scanning electron microscopy (SEM) was utilized to verify the miscibility of the low-density polyethylene matrix with additives. Figure 5 illustrates SEM images of freeze-fractured surfaces of pure LDPE and selected LDPE blends with tetracosane, paraffin wax, and paraffin oil films.

As expected, pure polymer and LDPE/poil systems presented a typical brittle fracture morphology. The surfaces of LDPE blends containing tetracosane or paraffin wax in an amount not exceeding 9% were also smooth and lacked noticeable heterogeneities, suggesting molecular dispersion of these modifiers in the polyethylene matrix (in the amorphous region between the lamellae) and the absence of phase separation. The occurrence of phase separation is shown in Figure 5f. In the LDPE/tetra9.5 system, tetracosane phase was represented as dispersed domains surrounded by the LDPE matrix. The shape was variable, mainly elongated, and the size changed from approximately 15 μm to a smaller size. A fairly homogeneous surface, without visible inclusions of modifiers, was also observed in a system, where DSC results indicated phase separation with domains rich in modifiers. In SEM images of the LDPE system containing 5.6% tetracosane (Figure 5e) or 9.1% paraffin wax (Figure 5i), the separated additive domains were difficult to detect, likely because they were smaller than 0.5 μm. These findings are consistent with our last study on high-density polyethylene and wax blends [20]. The behavior of polyethylene/additive blends is evidently influenced by multiple factors: the molecular weight of the additives, their compatibility with polyethylene, and the concentration of additives within the prepared systems.

**Figure 6:**
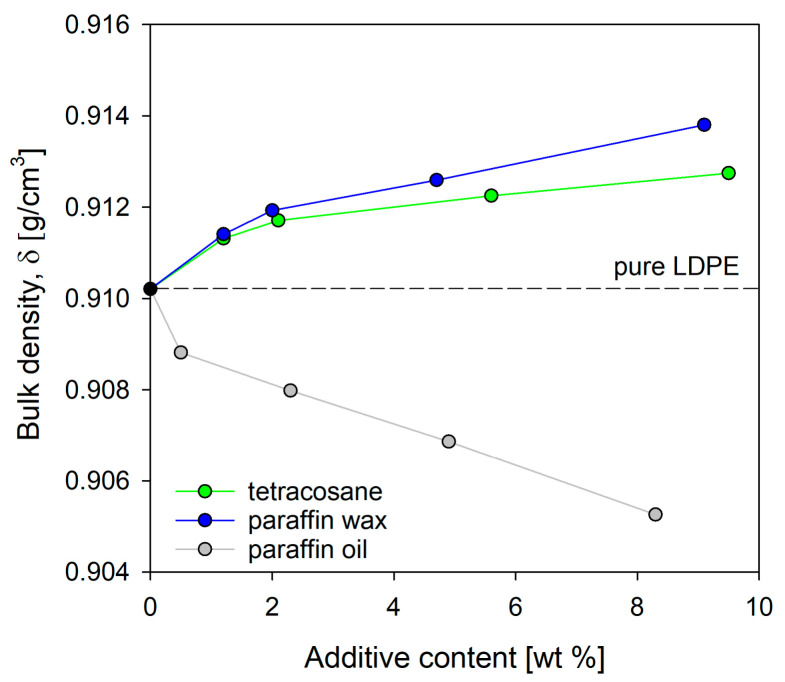
Bulk density for LDPE and its blends with modifiers (tetracosane, paraffin wax, paraffin oil).

The intermolecular interactions between polymer and additive can facilitate more efficient packing of the polymer chains. Consequently, denser packing of the chains indicates a reduction in free volume, which is crucial for the permeability coefficient [38,39]. The density of low-density polyethylene samples and its blends with tetracosane, paraffin wax, and paraffin oil was determined based on measurements of the equilibrium resting height of the samples in the density gradient column. In Figure 6, the bulk density values for both pure LDPE and LDPE/modifier systems are presented. At low concentrations of tetracosane and paraffin wax up to 2%, the density of the systems increased by approximately 0.16% and 0.18%, respectively, compared to pure LDPE. Since there were no notable changes in the degree of crystallinity of these blends (see Table 1), this increase should be attributed to enhanced chain packing. From a concentration of 4% to almost 10%, the density gradually increased, reaching the maximum value for LDPE/tetra9.5 and LDPE/pwax9.1 systems. In the case of these systems, the density increase compared to pure LDPE resulted from both an increased packing density in the amorphous regions and a higher degree of crystallinity.

It should also be noted that the phenomenon of phase separation occurring in systems did not cause a decrease in density. Phase separation promoted the formation of free volume, but the degree of crystallinity of tetracosane and paraffin wax, which was much higher than the degree of crystallinity of pure polyethylene, ultimately compensated for the excess free volume created by phase separation. It seems highly likely that for systems containing tetracosane or paraffin wax in an amount not exceeding 4%, the introduction of modifier molecules resulted in densification of the amorphous phase and, consequently, a reduction in free volume of polymeric material. Paraffin oil had the completely opposite effect on the density of the LDPE systems. Figure 6 clearly illustrates that as the concentration of paraffin oil in the polyethylene blend rose, the density sharply decreased. This means that the paraffin oil molecules did not compensate for the decreased molecular packing density in the amorphous layers resulting from their increased thickness (increase of LP values, Table 2). Bulk density measurements do not distinguish between changes in density due to variations in crystallinity and chain packing in the amorphous region. The local free volumes may arise from irregular molecular packing in the amorphous phase (static and preexisting holes) as well as from molecular relaxation of the polymer chains and terminal ends (dynamic and transient holes) [40,41].

**Figure 7:**
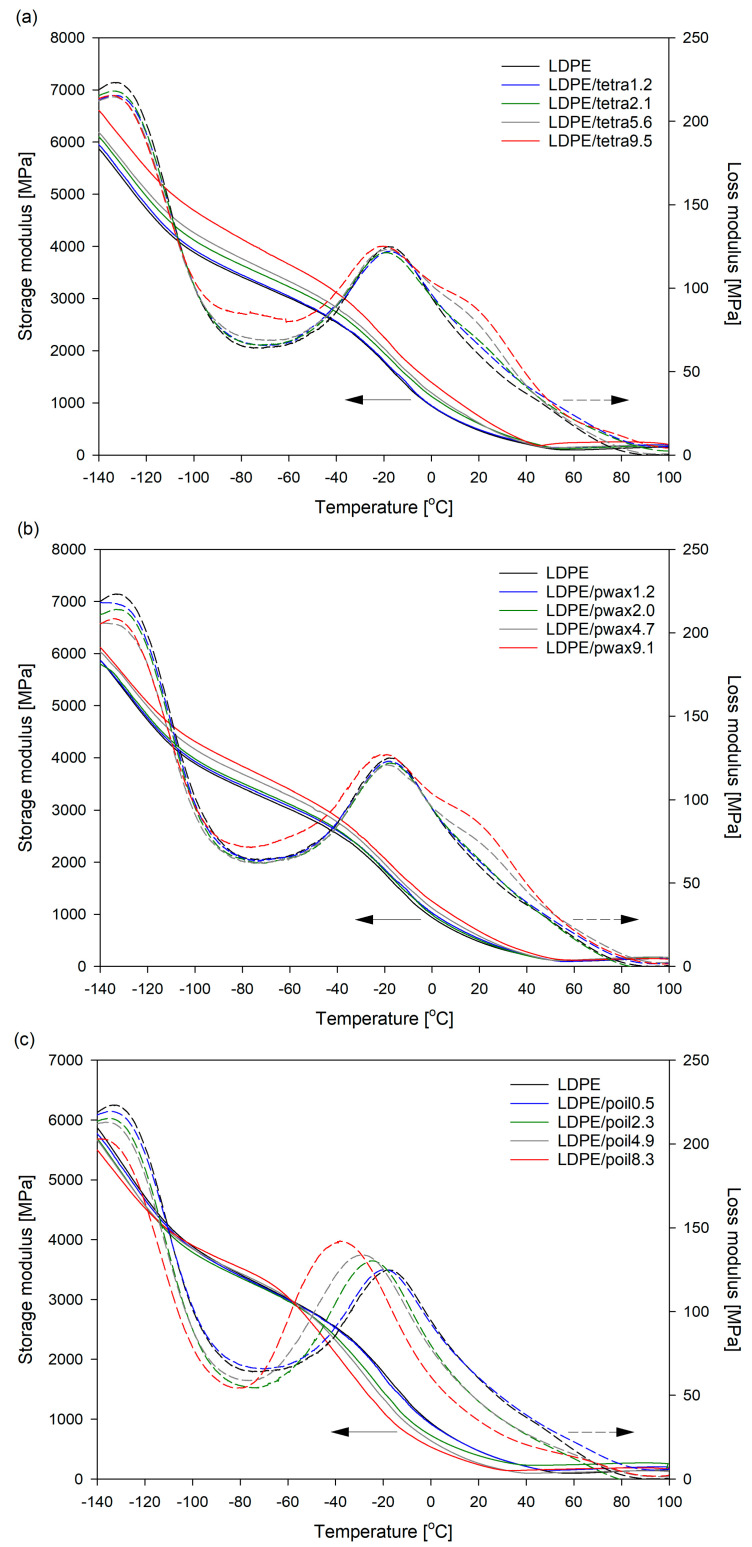
Temperature dependencies of storage (E′) and loss (E″) moduli for pure LDPE and LDPE blends with tetracosane (**a**), paraffin wax (**b**), and paraffin oil (**c**).

With this in mind, dynamic thermomechanical analysis (DMTA) was utilized to clarify the effect of incorporating additive molecules into LDPE on chain dynamics. The relaxation behavior is strongly influenced by variables that describe the crystalline–amorphous state, such as crystallinity, lamellar thickness, and amorphous layer thickness [42,43,44]. Thus, the incorporation of modifier molecules, which, as previously demonstrated, penetrated the amorphous phase, enabled alterations in the structural variables of such supermolecular structure. The mechanical relaxation spectra of pure low-density polyethylene and its blends with tetracosane, paraffin wax, and paraffin oil are shown in the form of storage and loss moduli in Figure 7.

Two E’’-maxima, referred to as β and γ, were distinctly noticeable in order of decreasing temperature: the β relaxation appeared between −60 and 20 °C, while the γ relaxation ranged from −145 to −90 °C. The β process is generally assigned to cooperative processes in the interlamellar amorphous phase [44,45,46]. It is postulated that β relaxation results from the relaxation of chain units in the interfacial region [46,47] and/or diffusional motion of branch points of segments (on both backbones and arms) in the amorphous matrix [9,48]. The γ relaxation is associated with small-scale motions within the amorphous component [44,49]. The γ process is also assigned to the glass transition of methylene sequence has have the same effect as the brittle–ductile transition [50]. This type of relaxation usually is explained by the molecular process of conformational changes in the amorphous region. Figure 7 clearly shows that the presence of modifier molecules affected both β and γ relaxation. The γ relaxation mechanism was related to the segmental motion of low-molecular-weight components, such as floating and cilia chains, in amorphous layers. Consequently, it appears reasonable to infer that modifier molecules influencing the molecular dynamics of these floating and cilia chains caused a slight shift in the γ relaxation position towards lower temperatures. A more important change from the point of view of barrier properties, caused by the inclusion of additives to LDPE, could be observed in the β relaxation region. The height of the relaxation peak was reduced by the introduction of tetracosane or paraffin wax in amounts up to 5.6% and 4.7%, respectively (Figure 7a,b). This indicates that the modifiers, by filling free volume, restricted the mobility of the polymer chains and increased the molecular packing density of the amorphous phase. In turn, better packing of the chains could affect the diffusion of gas molecules and ultimately alter the barrier properties of LDPE. The further increase in the concentration of these modifiers in the blend with LDPE unfortunately resulted in a slight increase in the height of the relaxation peak and a slight shift of its position towards lower temperatures. This behavior indicates a decrease in the density of the amorphous phase; however, in the case of the LDPE/tetra9.5 and LDPE/pwax9.1 systems, the increase in the degree of crystallinity compensated for these changes and, as a result, the bulk density of these systems increased (Figure 6). In the case of systems with paraffin oil (Figure 7c), a significant increase in the peak height and a shift in its position towards lower temperature values were recorded. The peak height increased with increasing paraffin oil content. This finding clearly indicates a reduction in the packing density of the amorphous phase as a result of the introduced additive. The results correlate very well with the results of density measurements.

The DMTA curves across the entire temperature range showed a typical decrease in the dynamic modulus E′, which was associated with the softening of the polymer matrix at a higher temperature. It is worth noting that the values of E′ in the case of tetracosane and paraffin wax consistently rose with their higher concentrations in LDPE systems. Such an effect could be attributed to the enhanced mechanical constraints imposed by the greater presence of additive molecules, which restricted the molecular mobility of LDPE. Tetracosane and paraffin wax (below 40 °C) immobilized the polymer chains, resulting in an increased modulus of the polyethylene matrix. Paraffin oil had a completely opposite effect on the E′ modulus values; namely, its presence caused the modulus values in the entire tested temperature range to be lower compared to pure LDPE. This was related to the physical state of this modifier (liquid state), which favored the softening of the polyethylene matrix.

The α relaxation process, observed in selected blends, was assigned to the motion of chain units within the crystalline phase [51,52]. According to numerous investigations, the α relaxation in the case of polyethylenes consists of two overlapping peaks designated as the α_1_ and α_2_ relaxations. The α_1_ relaxation is attributed to an intralamellar slip process (or grain boundary phenomena), and/or motion in the intercrystalline region, and the α_2_ relaxation is to intralamellar chain motion involving α transitional motion of chain segments along the c-axis within the crystal lattice [53,54]. The presence of α relaxation process in selected materials can be induced by a change in the degree of crystallinity of the LDPE matrix. A similar phenomenon, the appearance of α relaxation process, was observed by us in LDPE materials with a higher degree of crystallinity/thickness of crystals [55]. Overall, the presence of additives molecules does not seem to have much impact on the α relaxation. It can be seen that the position of the α peak for these samples was approximately 20 °C and was not influenced by the type of modifier or the increase in concentration in the mixture. Tetracosane and paraffin wax gave rise to weak marked α relaxation peak (probably α_1_), which, for pure LDPE and its blends with paraffin oil, was essentially absent.

The results discussed above indicate that incorporating modifiers such as tetracosane, paraffin wax, and paraffin oil into LDPE systems can alter both the molecular packing within the interlamellar amorphous regions and the crystallinity. The gas permeability was influenced by factors hindering the diffusion of gas molecules through the polymer film. Bearing this in mind, the introduction of additives into the polymer matrix, resulting in increased packing density in the amorphous regions of polyethylene (as evidenced by density and DMTA studies), also influenced the barrier properties of low-density polyethylene. To assess how the polyethylene/additive composition affects the final barrier properties, the transport properties of O_2_ molecules through selected LDPE blend systems were examined using permeation measurements.

**Figure 8:**
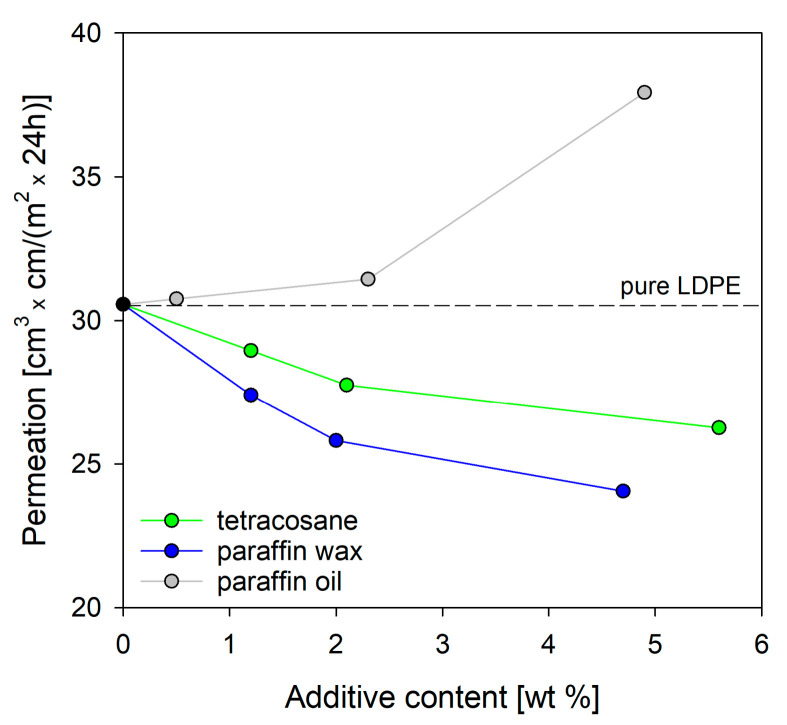
Oxygen permeability through films for pure LDPE and LDPE in systems with tetracosane, paraffin wax, and paraffin oil.

Based on the data in Figure 8, it is clearly visible that increasing the content of tetracosane or paraffin wax in the polyethylene blend enhanced its barrier properties. The highest increase in barrier properties was recorded for systems containing tetracosane and paraffin wax in amounts of 5.6% and 4.7%, respectively. Compared to pure polyethylene, this increase was 14% and 21%, respectively. Analyzing the results of thermal properties (DSC), bulk density (DCG), interlamellar distance (SAXS), and chain dynamics (DMTA), the question arose which factor turned out to be crucial in achieving the improved barrier properties in these systems. It seems that (i) the increase in the bulk density, (ii) the reduction in free volume, (iii) the increase in restriction to chain motion, and (iv) the slight increase in the degree of crystallinity contributed to this. Interestingly, blends of LDPE with tetracosane showed higher value of oxygen permeation than blends with paraffin wax. This could be explained based on the fact that LDPE/tetra5.6 had a higher free volume and a lower degree of crystallinity than LDPE/pwax4.7.

In the case of LDPE systems containing paraffin oil as a modifier, the barrier properties deteriorated. An increase in oxygen permeation was observed with increasing oil content due to the increased molecular motion in polymer chains. It is worth remembering that at the temperature of oxygen permeability measurement (23 °C), paraffin oil was above its phase change temperature and acted as a plasticizer. The obtained results are in good agreement with the data obtained for bulk density and chain dynamics.

## 3. Materials and Methods

### 3.1. Materials

Low-density polyethylene (LDPE) Lupolen 1840D with density of 0.919 g/cm^3^ was supplied by Lyondell Basell (Rotterdam, The Netherlands). Tetracosane with M_w_ = 338.65 g/mol, melting point (m. p.) at 49–52 °C and boiling point (b. p.) at 391 °C; paraffin wax with M_w_ = 436.84 g/mol, m. p. at 58–62 °C and b. p. at 322 °C; paraffin oil with M_w_ = 338.69 g/mol, m. p. at −24 °C and b. p. at 300 °C, were purchased from Sigma Aldrich (Sigma-Aldrich, Inc., St. Louis, MO, USA) and used as the low-molecular-weight modifiers. Hildebrand solubility parameters for LDPE, tetracosane, paraffin wax, and paraffin oil were 16.5 MPa^0.5^ [56], 17.3 MPa^0.5^ [56], 16.39 MPa^0.5^ [57], and 16.7 MPa^0.5^ [58], respectively. The differences in the solubility parameters, (δ_polymer_ − δ_modifier_)^2^, were therefore 0.64, 0.012, and 0.04, respectively. Based on the determined values, high compatibility of the additives with polyethylene was expected.

### 3.2. Blends Preparation

LDPE/additive blends, having various loading levels ranging from 0.4 to 10 wt%, were produced by melt mixing in a Brabender batch mixer at 180 °C for 5 min using a torque speed of 60 rpm. The content of modifiers has been estimated basing on TGA measurements (see Table 1). The neat LDPE was also processed under the same conditions to obtain a reference material. The prepared materials were then hot pressed at 180 °C using hydraulic press (90 MPa, 5 min) and quenched between metal blocks.

### 3.3. Characterization Methods

A thermogravimetric analysis (TGA) was conducted to estimate the modifier content using a Hi-Res TGA 2950 analyzer (TA Instruments, New Castle, DE, USA). Approximately 20 mg of each sample was heated at a rate of 20 °C/min, starting from room temperature up to 600 °C, under an air atmosphere.

The thermal analysis of samples was recorded through differential scanning calorimetry (DSC, Q20 TA Instruments, New Castle, DE, USA). A sample was heated at a rate of 10 °C/min from −100 to 180 °C. The enthalpy (Δ*H*_m_), peak melting temperature (*T*_m_), and crystallinity (*X*_C_) were obtained from the data of DSC curves. The calculation of the LDPE crystallinity was performed using the following equation:

(1) $$X_{C}=\frac{{∆H}_{m}}{{∆H}_{m}^{0}\times w_{LDPE}}\times 100\%,$$

where $w_{LDPE}$ is the mass fraction of LDPE in a sample, ${∆H}_{m}$ is the enthalpy of fusion, and ${∆H}_{m}^{0}$ is the enthalpy of fusion for 100% LDPE (293 J/g [59]).

Wide-angle X-ray scattering (WAXS) measurements were performed on a computer-controlled goniometer equipped with monochromatic Cu-Kα diffraction (λ = 0.154 nm) at a voltage of 30 kV and a current of 50 mA was used (Panalytical B.V., Almelo, The Netherlands).

The lamellar structure of analyzed samples was probed with use of 2-dimensional small angle X-ray scattering (SAXS). Technical details of the measurement method and long period calculations were presented elsewhere [60].

Fourier-transform infrared (FTIR) spectra were collected at room temperature on a Nicolet 6700 spectrometer (Thermo Scientific, Waltham, MA, USA) equipped with a deuterated triglycine sulfate (DGTS) detector. The technique of attenuated total refraction (ATR) was used for measurements. The spectra were obtained by adding 64 scans at a resolution of 2 cm^−1^.

The morphology analysis of the materials was performed on freeze-fractured samples using a scanning electron microscope (SEM), Jeol JSM 5500LV model (Tokyo, Japan). The fracture surfaces were sputtered with a thin layer of gold (approximately 20 nm) before observation.

Density was measured using a gradient column (DCG) constructed from a isopropanol and water solution in accordance with ASTM D1505 [61]. All the measurements were at 23 °C to prevent temperature-dependent density changes. Glass floats were used as calibration beads in the column to determine the local density. The details of the bulk density (*ρ*) calculations were described elsewhere [19].

Dynamic thermomechanical analysis was performed in a single cantilever bending mode using a Q-800 analyzer (DMTA, TA Instruments, New Castle, DE, USA). The dynamic temperature sweeps were conducted at constant frequency equal to 1 Hz within temperature scan ranging from −140 to 100 °C under a fixed deformation of 0.02% (heating rate of 2 °C/min).

Oxygen transmission rate (OTR) of the selected polymer films was analyzed using an XS/Pro-Totalperm permeability analyzer (ExtraSolution, Pieve Fosciana, Italy). According to ASTM D3985 [62], the oxygen transported through the material was monitored at 23 °C and 0% relative humidity. To reset any influence arising from different film thicknesses, OTR values were converted to permeability ([cm^3^ × cm/(m^2^ × 24 h)]).

The modifier from the blends with polyethylene was removed by extraction in a bath containing a (4:1:1) mixture of hexane, chloroform, and ethanol. The extraction process was carried out at room temperature for 5 days; then, the samples were dried and subjected to further studies.

## 4. Conclusions

Tetracosane, paraffin wax, and paraffin oil were selected as additives/modifiers to tune the barrier properties of low-density polyethylene (LDPE). The experimental results showed that the addition of a low-molecular-weight compound did not change the chemical structure and crystal form of LDPE. Moreover, the range of additive concentrations in the polyethylene matrix proposed in this work did not cause significant changes in the thermal properties of polyethylene. However, the content and type of the compound significantly determined the barrier properties of polyethylene. It was shown that polymer chain dynamics and free volume were the main factors affecting the barrier properties of LDPE. Tetracosane and paraffin wax present in the polymer matrix by a restriction in polymer chain motion, resulting in tortuous pathways for the diffusion of oxygen molecules, improve the barrier properties of the LDPE. The films prepared from LDPE/tetra5.6 and LDPE/pwax4.7 blends reduced oxygen permeability by approximately 14% and 21%, respectively, compared to films made of pure polyethylene. The results for LDPE/paraffin oil blends contrast with those for blends containing similar amounts of tetracosane or paraffin wax. The deterioration of the barrier properties in this case was attributed to the physical state of paraffin oil. By increasing the mobility of polymer chains, paraffin oil contributes to lower density, and the excess free volume that appears facilitates the flow of oxygen molecules.

## Figures and Tables

**Table 1:** Selected thermal properties of the low-density polyethylene and LDPE/modifier films by TGA * and by DSC ** analyses.

Sample	Sample Code	Modifier Content [wt %] *	*T*_mLDPE_ [°C] **	Δ*H*_m_ [J/g] **	*X*_CLDPE_ [%]
LDPE	LDPE	-	109.4	116.6	39.8
LDPE/tetracosane					
	LDPE/tetra1.2	1.2	109.3	118.1	40.3
	LDPE/tetra2.1	2.1	109.1	118.5	40.5
	LDPE/tetra5.6	5.6	108.7	122.6	41.8
	LDPE/tetra9.5	9.5	108.5	123.9	42.3
LDPE/paraffin wax					
	LDPE/pwax1.2	1.2	109.3	117.8	40.2
	LDPE/pwax2.0	2.0	109.2	118.8	40.5
	LDPE/pwax4.7	4.7	108.6	126.2	43.1
	LDPE/pwax9.1	9.1	107.7	129.7	44.3
LDPE/paraffin oil					
	LDPE/poil0.5	0.5	109.2	115.6	39.5
	LDPE/poil2.3	2.3	109.1	116.1	39.6
	LDPE/poil4.9	4.9	108.9	116.5	39.8
	LDPE/poil8.3	8.3	107.8	116.3	39.7

**Table 2:** Long period of pure LDPE and LDPE blends with tetracosane, paraffin wax and paraffin oil.

Sample	Sample Code	Long Period [nm]
LDPE	LDPE	11.7
LDPE/tetracosane		
	LDPE/tetra1.2	11.8
	LDPE/tetra2.1	11.9
	LDPE/tetra5.6	12.0
	LDPE/tetra9.5	12.0
LDPE/paraffin wax		
	LDPE/pwax1.2	11.8
	LDPE/pwax2.0	11.9
	LDPE/pwax4.7	12.0
	LDPE/pwax9.1	12.0
LDPE/paraffin oil		
	LDPE/poil0.5	12.0
	LDPE/poil2.3	12.4
	LDPE/poil4.9	12.7
	LDPE/poil8.3	13.0

## Data Availability

Data are contained within the article.

## Supplementary Materials

The following supporting information can be downloaded at: https://www.mdpi.com/article/10.3390/molecules29204950/s1, Figure S1: TGA curves of weight loss in air for pure LDPE and LDPE systems with tetracosane (a), paraffin wax (b) and paraffin oil (c).
