# Supplementary material for: Tuning Barrier Properties of Low-Density Polyethylene: Impact of Amorphous Region Nanostructure on Gas Transmission Rate
Source: Molecules. 2024 Oct 19;29(20):4950. doi: 10.3390/molecules29204950 (PMC11510230; doi:10.3390/molecules29204950)
Supplement: Supplementary file 1 [file molecules-29-04950-s001.zip › molecules-3254978-supplementary.pdf]

SUPPLEMENTARY MATERIALS

Tuning barrier properties of low-density polyethylene: Impact of amorphous region nanostructure on gas transmission rate

Marta Safandowska [1,*], Cezary Makarewicz[1] and Artur Rozanski [1,*]

[1] Centre of Molecular and Macromolecular Studies, Polish Academy of Sciences, Sienkiewicza 112, 90-363 Lodz, Poland
* Correspondence: marta.safandowska@cbmm.lodz.pl (M.S.); artur.rozanski@cbmm.lodz.pl (A.R.)

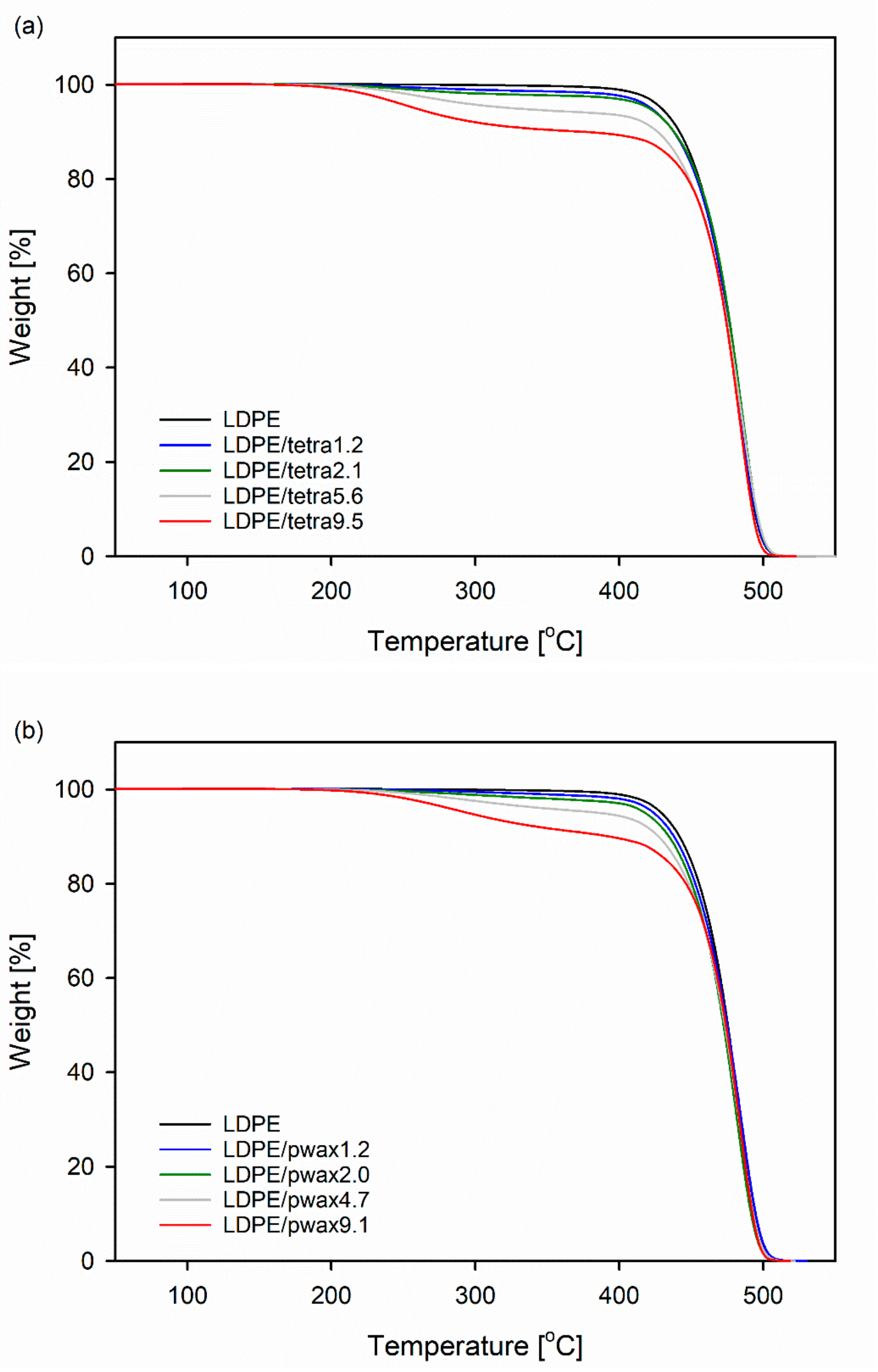
(a)
Weight [%]
Temperature [°C]
LDPE
LDPE/tetra1.2
LDPE/tetra2.1
LDPE/tetra5.6
LDPE/tetra9.5
(b)
Weight [%]
Temperature [°C]
LDPE
LDPE/pwax1.2
LDPE/pwax2.0
LDPE/pwax4.7
LDPE/pwax9.1

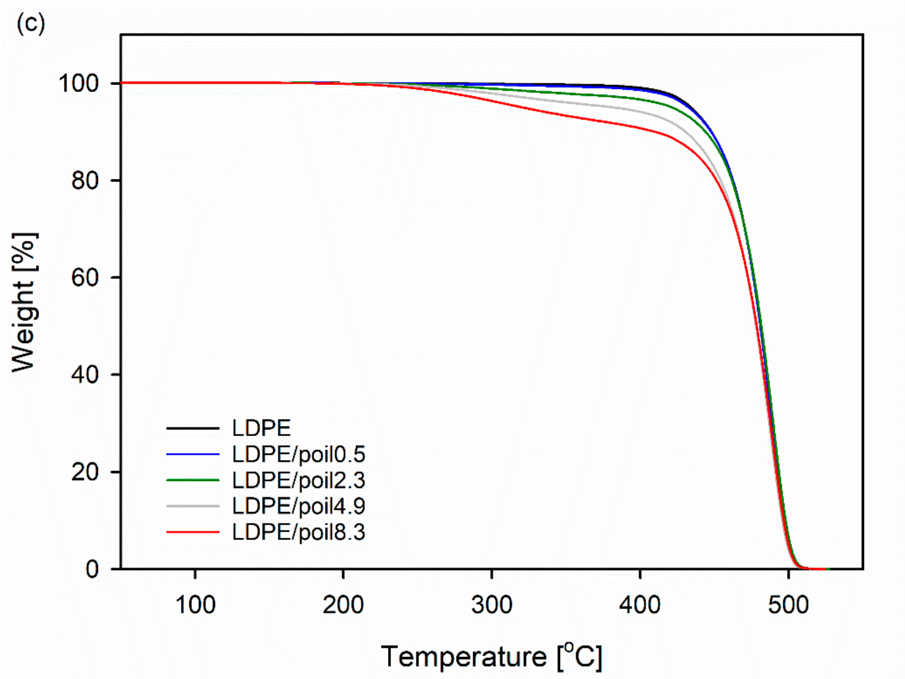

Fig. S1. TGA curves of weight loss in air for pure LDPE and LDPE systems with tetracosane (a), paraffin wax (b) and paraffin oil (c).
